# “There is a hell and heaven difference among faculties who are from quota and those who are non-quota”: under the veneer of the “New Middle Class” production of Indian public universities

**DOI:** 10.1007/s10734-022-00932-7

**Published:** 2022-09-28

**Authors:** Nandita Banerjee Dhawan, Dina Zoe Belluigi, Grace Ese-Osa Idahosa

**Affiliations:** 1 School of Women’s Studies, Jadavpur University, Kolkata, India; 2 Queen’s University Belfast, Belfast, Northern Ireland UK; 3 Nelson Mandela University, Gqeberha, South Africa; 4 Centre for Education and Social Justice, University of York, York, UK; 5 Centre for Social Change, University of Johannesburg, Johannesburg, South Africa

**Keywords:** Quality, Gender equality, Caste discrimination, Academic staff, Leadership, Sustainable development goals, India

## Abstract

The university is a highly politicized and fractious realm for students and academics. Amidst trade-offs between the processes of massification, democratization, commodification, and globalization, the question of transformation *for* sustainability has become crucial to the social good(s) of higher education. This paper considers academic citizenry within Indian public higher education — a context where the increase in the enrollment of first-generation students and female students, due to affirmative action policies, has not substantially translated into altering the composition of academic staff. Informed by a mixed-method study conducted in 2019 with the participation of academics and those in leadership positions at four higher education institutions, we found that the enactment of such policies was operationalized for the production of the “New Middle Class” by universities. Of concern is that neither the representation nor the participation of academics who are women, “lower” castes, or minorities meets the mark of just, inclusive institutions. Despite the rhetoric of inclusiveness and development, the implementation of related policies clothe subalterns with the veneer of the intellectual class, permitting access on condition that sociocultural identities are concealed, and the hegemonic status quo maintained. Terms such as “quality” and “equality” function as tools for social control rather than serving social justice, where assertions of caste identity and resistance are simultaneously repudiated and misrecognized.

## Introduction

We begin this paper by discussing a case of discrimination experienced by an academic in September 2020 in West Bengal, a region of India often lauded as more liberal and intellectual than the majority of the country. We do so because it brings to the fore the continued gendered and casteist marginalization and victimization of women and those groups deemed “lower” within the social hierarchies of Indian higher education institutions (HEIs).

When a professor of History from Jadavpur University, Dr. Maroona Murmu, shared on social media her concerns that examination practices were placing students’ lives at risk during the COVID-19 crisis, casteist slurs were posted on Facebook by members of the student communities of Kolkata. One wrote that.

It’s not about lagging one year but about how some unqualified and incompetent people take undue advantage of the reservation system and their caste is now helping them be successful, while the deserving lag behind forever. Our parents are stepping out, taking a risk every day to get us food, while some are sitting at home and getting paid for doing nothing. (Ghosh, 2020, n. p.)

Words such as “unqualified”, “incompetent” and “worthless” were used to describe the professor, with accusations that she was taking “undue advantage” of what was constructed as her “quota professor” identity. As the reader will notice in this paper — which offers an analysis of data collected in four universities in 2019 for a study on the politics of academic citizenry in Indian higher education — such a deficit discourse was echoed by many of the academics who participated. One such example of the binaries within this discourse was revealed in a statement made by a female associate professor (P15), which we chose for this paper’s title:

There is a hell and heaven difference among faculties who are from quota and those who are non-quota.

Dr. Marmu was hounded by over 1800 online trolls following that one social media posting. This was not the first of such experiences for her. She was quoted within the news media as being “appalled, but not shocked as she had been at the receiving end of casteist and racist slurs almost on a daily basis” within university spaces (Ghosh, 2020, n.p.). The social media thread is impactful for bringing to the surface the tacit pattern often denied by dominant in-groups, of “academic untouchability” where academic worth continues to be measured by one’s social position in the casteist hierarchical order. One of the first Adivasi academics in the social sciences and humanities at that university, Dr. Murmu has taken a proactive position on the matter in her academic citizenry and public intellectualism. She asserts it is her “academic responsibility to speak up” (quoted in Ghosh, 2020, n.p.) and, as such, she lodged a formal complaint at the police station under the Scheduled Castes and Tribes Prevention of Atrocities Act 1989, and has engaged in public discourse on such issues (see for instance Bhattacharyya, 2020; Roundtable India, 2020; Ghosh, 2020; Kar, 2020; Chowdhury, 2020).

Earlier that year, in a presentation at a colloquium about higher education organized by the first two authors of this paper at Jadavpur University,^1^ Dr. Murmu (2020, n.p.) had shared that.

As soon as the Dalit or Adivasi person enters the academic space, institutionalized discrimination begins to repress them. They are made to understand that these spheres do not belong to them and they are to be always marginalized… [the] structure of the mental harassment, humiliation is so powerful that it curbs the wish to resist.

She presented her counter-story of higher education, beginning with the hopes of her upper caste mother who had fallen in love with her Adivasi colleague while at university and later married in a rejection of caste-endogamous norms. With the cost of such difference extended inter-generationally, Dr. Murmu was made to pay the price for what can be seen as her mother’s transgressions of that deemed “legitimate” in marriage. Dr. Murmu’s treatment as “an exhibited strange object” in school extended through to postgraduate study where she was actively discouraged by her teachers from researching the “high culture” of classical music in eighteenth century Bengal because, as a “tribal”, she was constructed as not “qualified” enough. This deficit positioning from casteist discrimination has continued in her professional academic life, in incidents with students such as that recounted above, and with fellow academics who have accused her of using her caste identity to gain popularity and attract attention. In early 2020, her story of resistance as a marginalized academic was visualized in an art-based research project (Belluigi & Meistre, 2021; Wilby, 2020).

One of the great leaders and thinkers around the question of the so-called “untouchables”, Dr. Bhimrao Ramji Ambedkar, argued that inter-caste marriage was an act of violating caste endogamy such that men and women of different castes might act on their desires and their respect of one another (Rao, 2012).^2^ Similar hopeful conceptions of interactional dynamics on university campus spaces and through processes of enlightenment in learning in higher education, proliferate. However, the Janus face is that those positioned within such “openings” face the might of those invested in retaining their power through exclusion, marginalization, and delegitimation. Such dynamics were evident in India during upper caste protests after the Mandal recommendations^3^ in the 1990s (Chakravarti, 2002) and their persistence continue to be revealed in the micro- and macro-aggressions meted out to those, such as Dr. Murmu, in current times. Such insights point to how institutions of higher education and endogamous marriage can act in ways which are mutually constitutive in (re)enforcing the hegemonic social order. They remind us of how paradoxical ideographs characterize the public university, in part because the institution extols openness and equality while simultaneously reaffirming hierarchies and distinction.

Individual, collective, and institutional cultures of reflection and critique have been offered by researchers and practitioners as central to practicing radical commitments to democracy (Archer, 2007; Arunima, 2017; Belluigi, 2020; Bhushan, 2019; Deshpande, 2016; Kumar, 2016; Nair, 2017; Visvanathan, 2000). This is in an attempt to trouble and redefine the relations between intellectual activities within the university and its “outside”, such that the university is enabled to productively utilize its two important functions — social formation and knowledge formation — for social justice (Brennan & Naidoo, 2008, Kumar, 2016; Belluigi, 2020). Attempts at slow, incremental change to the social institution of the university have been explored across the world, often through such policies of “affirmative action”, “positive discrimination”, or what is colloquially known in India as the “quota system” or “reservations”. Many of those from critical traditions question the impact of such approaches to change for addressing local and global injustices, and the costs on lost generations and knowledges. One of the questions posed is how “access” for student entrants relates to myths of social mobility, and another, how disruptions to the status quo of the university itself are deferred through the notion that “pipelines” will inevitably lead from marginalized student entry to democratized academic citizenry (Brim, 2020; Reay, 2018; Xu, 2020) and to a diversified staff composition. However, when dehistoricized understandings of the need to transform higher education are further emptied of notions such as justice, there is increased danger that the social groups previously denied the right to education and to knowledge production will continue to be misrecognized and cast as deficit or lacking. The machinations of domination within the system will be maintained. Questions have also been posed about why the burden of dismantling systemic oppression and injustice does not fall on those whom have benefitted from what their ancestors created, but on those who bear the markers of difference (Belluigi, 2020).

As with such critical traditions, in this paper, we draw attention to the “dark” sides (Bengsten & Barnett, 2016) of interactional dynamics within the academy, to emphasize that the *regulation* of access for the inclusion of socially marginalized groups cannot alone lead to equitable relations, participation, and systems within higher education. We do so by firstly offering a historical narrative of the regulation of access in the public university in India, indicating that it has always already been a politicized space, in challenging conceptions of racial difference (Datla, 2013; Seth, 2015), as a site of nationalist and revolutionary agitation during colonial times (Carnoy & Dossani, 2013; Singh, 1991), and in the attempts to address gender and caste inequalities in independent India (Nair, 2017).

## The regulation of access

The expansion of higher education in independent India led to major changes in the texture of universities, particularly in its negotiations of the predominantly male, upper caste, middle class nature of the student population. The demography of the student populations of the Indian university steadily changed due to government-stipulated reservation (the “quota”) for those castes previously marginalized — specifically the “Scheduled Castes” (SC), “Scheduled Tribes” (ST) and “Other Backward Classes” (OBC).^4^ The increase in such “diversity” in the student body has posed important challenges in terms of facilities, infrastructures, and attitudes to massification, which were arguably not adequately addressed in practice, policy, or research. Least successful has been policy and practice in the recruitment and retention of academic staff within such categories as we discuss in this section.

The Gross Enrollment Ratio (GER) of students rose from 8 to 27.1% between 2001 and 2020, achieved through the 1043 universities in India, of which there are 386 state public universities, 48 central public universities, and 135 institutions of national importance (AISHE, 2020). The most recent snapshot is provided by the AISHE Report (2019–2020) which does not reflect intersectional nuances, such as how gender works with other structures of discrimination and oppression, such as those related to caste, religion, and rural/ urban location. Such intersectional considerations are of importance when analysing the implications of the recent New Education Policy (2020) which posits 2035 as the deadline for a GER of at least 50%, because these interlocking effects have been found to produce greater disparities for women than for men across the same social groups at entry level in universities (John, 2012; Shaguri, 2013).

Male students constitute 51% and female students constitute 49% of Indian students, with Gender Parity calculated as 1.05 for SCs and 0.97 for STs and 1.01 for the category “all”. The most recent composition indicates that OBCs comprise 37% of the total student population in Indian higher education, with SCs and STs comprising 14.7% and 5.6%, respectively (AISHE, 2020). However, GER varied widely across these categories with SCs and STs having GERs of 23.4% and 18%, respectively, in comparison to all who had a GER of 27.1%. In terms of gender distribution, the male and female student population of SC has a GER of 22.8% and 24.1%, respectively, and ST has a GER of 18.2% and 17.7%, respectively.

It is at the level of academic staff composition in higher education in India, the subject of this paper, where the inclusion of women, lower castes, and minorities falls far below the mark of equality despite the supposed pipeline from student quotas. Out of the 1,503,156 academics in higher education institutions (HEIs) in 2019–2020, 57.5% were men and 42.5% are women. However, gendered academic hierarchies emerge when employment positions are shown, as in Tables 1 and 2.

**Table 1:** Academic staff in Indian higher education institutions, categorized according to academic position (AISHE 2019–2020)

Category	Distribution	Female	Male
Professors	9.3%	28%	72%
Associate professors/readers	10.7%	37%	63%
Assistant professors/lecturers	68.1%	43%	57%

**Table 2:** Levels of professorship in Indian higher education, by gender binaries across institutional types (AISHE 2019–2020)

	Professors		Associate Professors		Assistant Professors	
	Male	Female	Male	Female	Male	Female
Central universities	78%	22%	74%	26%	70%	30%
Institutes of National Importance, including Institutes of Technology (IITs) and Institutes of Management (IIMs)	84%	16%	81%	19%	78%	22%
State public universities	75%	25%	67%	33%	60%	40%

The gendered composition of academic staffing emerges in the different institutional types, as indicated in the table below.

Further disparities emerge within the social stratification between institutions. The HEIs which are seen as more elite, the “Institutes of National Importance”, have a very low percentage of academics from SC and ST categories in their staff composition. According to the data shared by the Union Ministry of Human Resources Development, Government of India in 2019, out of the 6043 faculty members, at the 23 IITs, 149 were SCs and 21 STs (Wire, 2019). Data collected from 2016 to 2018 revealed that of 642 faculty members at 13 IIMs, only 4 were SC, 1 ST, and 17 from OBCs (Joshi & Malghan, 2018; Wire, 2017).^5^ Intersectional analyses of gender and social group have not been made publicly available.

Such figures lead us to critically reflect on “why we are witness to possibly more than one lost generation of students who are yet to take their place as teachers” (Nair, 2017, p. 36) despite the claim that affirmative action policies have been implemented. Some may argue that this is due to economic constraints, which both severely affect the future aspirations of those from historically disadvantaged sections of the society, and impact on the declining financial investment in public universities, fee hikes, stagnant scholarship opportunities, and the rapidly growing private sector. However, these claims should not be utilized to divert attention from addressing the political internal dynamics within higher education which continue to thwart the implementation of affirmative action policy as a social justice intervention.

### The “Mandalization of politics” and the rise of the “New Middle Class”

Scholars have analysed the historical and political processes that, from colonial times, have enabled the privileged upper castes to think of themselves as “casteless”. This is despite being inheritors of property, prestige, and power even as they continue to assert their moral and political leadership in independent India through their support for economic liberalization (Alam, 1999; Bhatt et al., 2010; Deshpande, 2013; Dirks, 2001). While caste may have seemingly become irrelevant for those from the upper caste, through being overwritten by their professional identities of choice, it became hypervisible for the lower castes by rendering invisible all other possible identities. Deshpande (2013) argues that the key to understanding caste was the notion of “castelessness” and “naturalization” of the upper castes as they assumed the mantle of the legitimate inheritors of modern forms of capital which included property, credentials in higher education, and lucrative professions. However, this presumptive castelessness did not require the upper castes to give up their caste in reality; they were assured of being presumed to be casteless as long as they did not choose to invoke their caste explicitly. Caste therefore was no more recognized as a source of privilege, either constitutionally or legally; it was recognized only as a source of vulnerability (Deshpande, 2013, p. 37). This became especially evident in the 1980s, a decade marked by newly exploding caste identity and consciousness, as lower caste groups attempted to take strong steps to gain political power.

What has become known as the “Mandalization of politics”^6^ in the 1990s radically altered the social bases of politics in India. The category of the OBCs was added to the existing “reserved” categories, comprising of SCs and STs, with the administrative duty on public institutions to implement the reservations policy, as regulated steps towards realizing their political and social status as new socio-economic groups. Sheth (1999, p. 2504) refers to this change in caste consciousness as a de-ritualization of caste in post-colonial India, which entailed the “delinking of caste from various forms of rituality which bounded it to a fixed status, an occupation, and to specific rules of commensality and endogamy” so that “the hierarchically ordered strata of castes functioned as horizontal groups, competing for power and control over resources in society”. With the Mandal moment, the fiction of the unreserved “general” category being the all-inclusive universal category was debilitated. Instead, the general category became a euphemism for the privileged upper castes who constitute a minority section of the Indian population (Deshpande, 2013). The implementation of a quota system in the recruitment of Public Services and Central Services was intended to increase the recruitment of the so-called backward castes with an inevitable proportionate fall of share of those from the “forward” upper castes. Thus, reactions to Mandal Commission recommendations included strong protests, such as those of “unmarked casteless” female students who in some cases held placards on which was written “we don’t want unemployed husbands” (Chakravarti, 2002, p. 1). The implication was that endogamy continued as a dominant norm in terms of reproduction of the caste system with the message on the placards reflecting the anxieties for the futures of upper caste girls which would be supposedly adversely affected by being possibly deprived of upper caste Indian Administrative Service (IAS) husbands. The related implication was that it was unimaginable that those, from the backward castes who would occupy these privileged jobs due to reservations, would break through the inviolable barriers of caste to be potential husbands for these young women (Chakravarti, 2002; Dhareshwar, 1993).

Subramanian (2019) outlines how technical knowledge, which was the purview of lower caste artisans in the nineteenth- and twentieth-century colonial order, gradually transformed into symbolic of state power, socio-economic development, and upper caste status in independent India. The establishment of the original five Indian Institutes of Technology (IITs) in the 1950s, and the emergence of engineering as a white-collar profession, redefined the relationship between technical knowledge and caste formation. This was because the lower caste artisan was sidelined in favour of upper castes aspiring towards a new profession. Such moves reveal the complex relationship between caste and merit where the democratic idea of preserving “merit” in the institutions feeds into the reproduction of inequality both within and outside HEIs. Institutions of National Importance, such as the IITs and Indian Institute of Science (IISc), with their predominantly upper caste composition are “gated universes” which claim to be “natural inheritors of scientific practice” (Thomas, 2020, p. 1), thereby constructing their individual identities as casteless as they simultaneously resist and consistently oppose affirmative action policies in the name of preserving merit (Subramanian, 2019; Chadha & Achuthan, 2017).

The post-Mandal phase again revealed the delusions of the self-perceived transcendence of these middle classes from caste-consciousness. The perceived threat from the reservations for lower castes increased their social anxieties and urgency to realize the preservation of their privilege. While they had no option but to make some space for the entry of different sections of the lower castes, large sections of the Hindu middle class were involved in “elite revolts” in response to the upsurge of the lower castes and in opposition to caste-based reservations (Corbridge & Harriss, 2000; Fernandes & Heller, 2006; Krishnan, 2017; Upadhya, 2016). Sheth (1999, p. 2508) referred to this process as the “classisation of caste” where caste acquired “new economic interest and a political identity. Its members now negotiate and own larger and multiple social and political identities”. As a result, the new socio-economic formations of the General category (upper castes), the OBCs (backward classes), the SCs (Dalits), and the STs (the tribals), competed for control of resources in society, with notions of upward social mobility motivating people of all castes, collectively as well as individually. The upper castes were advantaged by their control over resources of their traditional higher status, with a small section of lower castes capacitated to address their disadvantage through state policies of affirmative action (Louis, 2005), which led to a kind of fusion between the old status system and the new power system.

Sheth (1999) characterized this as the “New Middle Class” (NMC), including the adjective “new” because of its novel emergence from the disintegration of the caste system. Fernandes and Heller (2006, p. 497) described the NMC as a “class-in-practice” which is a “tangible and significant phenomenon”, one whose “boundaries are constantly being defined and tested” forged at the intersection of liberalization and a context marked by the political assertiveness of the lower castes. These scholars considered the NMC as “socially diverse” and “not a unified category”, where members of different castes could enter this class on having acquired modern education, employment within non-traditional occupations, commanding higher incomes, and political power. Both the politics and everyday practices, through which the NMC reproduces its privileged position, play an important role in its reproduction. The dominant fractions of the NMC have continued to reproduce their hegemonic positioning through patriarchal institutions of caste, marriage, religion, and education. Such mechanisms as endogamy^7^ and the command of the English language have been significant instruments in shaping resources and influencing their everyday practices (Dhawan, 2010, 2011; Fernandes & Heller, 2006).

### The “New Indian Woman” at the intersection of caste and gender

When the Indian economy was opened to foreign investment, it was advantageous for a narrow segment of the NMC, especially its managerial-professional segment out of which a small fraction consisted of urban-educated middle class women. The Indian state hailed this “new Indian woman” as the empowered symbol for India and deployed the optics of her public presence to proclaim it was a nation free from gender discrimination. She enthusiastically embraced the emergence of the nation into the global arena by becoming a consumer and pursuing a successful career. Claiming all the while to be modern, unmarked, and universal Indian citizen, her politics was revealed when protesting against the Mandal Commission recommendations (Dhawan, 2010; Fernandes, 2004; Sen et al., 2011). The repeated invocation of the figure of the new Indian woman in the political construct of the NMC not only failed to engage in solidarity with the struggles faced by poor, lower caste, subaltern women but also obfuscated those injustices. The upper caste, middle class new Indian woman belonging to the General category occupied the figure of the “ideal” representative of the NMC. This then became dependent on the figure of the subaltern woman, on the other hand, who as the “other” could no more be distanced by NMC illiberalism; her assimilation became vital to the articulation of the NMC as inclusive and expansive for modern independent India at that historical conjuncture (Bhatt et al., 2010). However, the entry of the subaltern woman into the domain of higher education paved the way for anxieties of her competing for higher income jobs and the possibility of upward social mobility.

While the institution of higher education has been somewhat regulated, there are no constitutional obligations to question nor disrupt endogamy as a dominant norm which reproduces the caste system (Chakravarti, 2002; Krishnan, 2014; Nisbett, 2013; Sancho, 2013, 2015). Thus, endogamous marriage as a public and political act has continued to perform the functions of an influential patriarchal institution which ensures the hegemonic caste order by regulation of female sexuality. Similarly, moves continue institutionally to prevent lower caste men from sexual access to upper caste women. Thus, caste and gender are mutually constitutive categories where women are positioned to play a crucial role in enacting the boundaries of caste, through which both caste hierarchy and gender inequality are maintained. Scholars further propose complicating gender as an analytical-political category — to move beyond the act of rescuing or empowering “women” from the gender binary, to where the differences *between* women are acknowledged as being produced from the different placements they occupy and are positioned within in the hierarchies of domination and resistance (Chakravarti, 2002, Chadha & Achuthan, 2017, Mohanty, 2003).

As the “classisation of caste” resulted in both upward and lower castes competing for access to higher education for credentials which promised to better secure higher incomes, it has become imperative to explore the ways in which gender and caste mutually inflect each other in universities, and the possibility of considerably altering the paradigms of social formation and knowledge formation. With the university becoming a space of “reshaped socialities” (Nair, 2017, p. 35), a question we ask of ourselves as researchers is “how does one study the (public) university for social justice^8^ transformations?” (Belluigi, 2020).

## Research methodology

The paper is an output of a research project which focused on the question of the validity of higher education as a social institution in terms of its fitness for purpose to drive substantive changes in the interests of the most marginalized, (un)privileged, and (mis)recognized in the two Middle Income Countries of India and South Africa.^9^ The project raised various questions about sustainability in higher education and how the meso- curriculum shapes academic citizenry, gender equity, institutional leadership, and the politics of participation in both contexts (see for instance, Dhawan et al., 2020, 2021; Belluigi et al., 2020). This paper focuses specifically on findings generated from the participation of academics and leadership from four institutions in India. Out of more than 400 public universities in India, we purposively selected an old and large university of high national ranking (UI1), a previously old college upgraded to university status (UI2), an institute of technology of national importance (UI3), and an institution of eminence (UI4).

We adopted a mixed-method approach to study these institutions in two phases sequentially. In the first phase, data was generated from 136 participants of the 4 institutions who responded to a questionnaire of 34 questions in total, 25 of which were close-ended and 9 were open-ended. When the initial online response rate proved inadequate, despite the encouragement of snowball sampling, in-person recruitment was undertaken. The responses in this phase were analysed for statistical signification. In the second phase, in-person appointments were made with participants via email for semi-structured interviews, with snowball sampling encouraging further recruitment. The in-depth interviews were conducted face to face and lasted approximately from 60 to 90 min. COVID-19 restrictions prevented data generation from UI2; however, 37 participants were recruitment from 3 institutions (UI1, 3, and 4) as outlined in Table 3. These included 30 members of academic staff and 7 of those we refer to as “leaders” within this paper, who were in the assigned leadership positions of dean or higher in the university hierarchy at the time of interview. Further indications of the demographics of the participants are indicated below; however, their disciplinary background has been ethically obscured. The disciplinary spread of participants included those within the Arts, Humanities, Social Sciences, STEM, Health and Life Sciences, and Women’s Studies. Most were from the Arts and Humanities (11), and the least from Health and Life Sciences (2). Of the 15 female academics participating, 8 were either tenured in centers or schools of Women’s Studies or were designated to promote gender mainstreaming from within departments in the disciplines of Arts, Humanities and Social Sciences in which they were situated. For the sake of confidentiality we have extended the term “gender persons” (Henderson, 2019)^10^ to include all of those 8 participants.

**Table 3:** The composition of participants according to institution, position, gender, and social group

Participant Codes	Institution	Position	Gender	Caste
P1	UI1	Academic staff	Male	General
P2	UI1	Academic staff	Male	General
P3	UI1	Academic staff	Female	General
P4	UI1	Academic staff	Male	General
P5	UI1	Academic staff	Female	General
P6	UI1	Gender person	Female	General
P7	UI1	Gender person	Female	General
P8	UI1	Gender person	Female	General
P9	UI1	Leader	Male	General
P10	UI1	Leader	Male	General
P11	UI3	Academic staff	Male	General
P12	UI3	Academic staff	Male	General
P13	UI3	Academic staff	Male	General
P14	UI3	Academic staff	Female	General
P15	UI3	Academic staff	Female	General
P16	UI3	Academic staff	Female	General
P17	UI3	Academic staff	Male	General
P18	UI3	Academic staff	Male	General
P19	UI3	Gender person	Female	General
P20	UI3	Gender person	Female	General
P21	UI3	Leader	Male	General
P22	UI3	Leader	Male	General
P23	UI3	Leader	Female	General
P24	UI4	Academic staff	Male	SC
P25	UI4	Academic staff	Male	SC
P26	UI4	Academic staff	Female	General
P27	UI4	Academic staff	Female	General
P28	UI4	Academic staff	Female	General
P29	UI4	Academic staff	Male	General
P30	UI4	Academic staff	Female	General
P31	UI4	Academic staff	Male	General
P32	UI4	Academic staff	Male	General
P33	UI4	Gender person	Female	General
P34	UI4	Gender person	Female	General
P35	UI4	Gender person	Female	General
P36	UI4	Leader	Male	General
P37	UI4	Leader	Male	General

Of the 30 academics not in assigned leadership positions, 13 were male and 17 were female; they included 17 professors, 7 associate professors, and 6 assistant professors. All 7 leaders were senior professors, composed of 6 men and 1 woman, with 1 director, 2 pro-vice chancellors, 2 deans, and 2 members of their institutions’ board of governors. In terms of social group, 35 participants belonged to General category, with only 2 academics, both from UI4, belonged to the “Scheduled Caste”.^11^ We realize that the respondents to the interviews, whose data informs this paper, are predominantly Hindu and from the General category. There was only one Muslim respondent. While limiting our access to insights into the lived experiences of those marginalized, it provided us with the opportunity to examine the complex layers of the everyday practices and strategies employed by those regarded by scholars (discussed above) as claiming to be casteless democratic citizens and their relation to the preservation of meritocracy in Indian HEIs. However, we do intend to engage in further research with academic citizens of the marginalized social groups and religions.

Questions posed during the in-depth semi-structured interviews were designed to elicit insights of the participants’ positionality, attitudes, and perspectives on transformation in higher education, and to shed light on the politics of participation within their institutions. In addition to questions about the history and cultures of their institutions, policy, and practice interventions, the interviews included explicit questions about whether the choice and autonomy of the participants enabled them to implement their beliefs about transformative change in higher education in the realms of teaching, research, and engagement with community. Selected examples of such questions included the following:

- What do you understand by transformation for sustainability (TFS)?
  - In what ways do you see relations to Gender equality (SDG 5), Strong institution for peace and justice (SDG 16), and Quality education (SDG 4)?
  - Which structures and cultures in your institution do you feel need changing to attain the TFS?
  - What are the markers of difference (including gender, race, caste/ethnicity, religion, and ability) in your institution?
- Do you generally feel YOU have the power to bring about these changes?
  - From your own experience, what enables/limits such power?
- Following our discussion on the importance of TFS, can you discuss your choice/agency and autonomy in your teaching, your research, and when you engage as an academic/researcher within and outside academia?

For those in leadership positions, additional questions included the following:

- How can universities deal with issues of social justice and serving marginalized communities?
- Does your university focus on it? If yes, how?
- What kind of challenges do you face?

Questions such as these were designed to enable participants to reflect and to share, for those with such critical consciousness, the challenges faced to enacting their beliefs, and the relevant costs involved when their capacity to tackle these challenges was threatened or constrained. We further tried to comprehend how participation was constituted in relation to markers of sameness and difference including those of gender, caste, religion, and various abilities. From those in leadership positions, we additionally enquired about tensions between existing policies and institutional initiatives, and their imaginaries of transformation as leaders. We hoped that by creating space for their reflections on their agency as leaders, and their perspectives on politics of belonging of marginalized groups in the institutional space, we would be able to comprehend their notions of social justice transformation in HEIs.

Within the larger framework of the rise of the NMC, feminist lens enabled understandings of the insider perspectives of the academics and leaders, and their participation in the processes of incorporation of subalterns in university spaces. The “Findings and discussion” section below includes excerpts of the participants’ qualitative responses to provide the reader a tangible sense of the tacit aspects and discourses of the politics of participation in these institutions. In undertaking the analysis and interpretation of the data, the purpose was not to produce generalizable knowledge or claims representative of all academics in Indian HEIs. Rather, the findings allude to a broader set of recognizable features in the discourses about the interactional staff dynamics within the larger, fraught context of struggles related to the democratization and massification of higher education.

## Findings and discussion

In this section, we discuss how tensions about conceptions of quality and equality underpinned participants’ narratives which, when probed, revealed intersecting and, at times, competing positioning by respondents. We then discuss how these seemed beset by denial, misrecognition, and blame shifting about the conditions of suffering faced by marginalized academic citizens.

### Deficit capital: tensions in quality and equality discourses

Tensions in the discourses of merit, equity, and equality underpinned many participants’ narratives. There was an overwhelmingly strong support expressed by those of the General category for meritocracy. In their responses they either implied or explicitly asserted that there should be a removal of social barriers from the criteria for entry to higher education and the ending of affirmative action policies. The continued indicators of casteism, in terms of awarding gaps in students’ assessment results, were constructed as politicized fictions rather than actual.

A female assistant professor from UI4 (P28), the Institution of Eminence, was deeply concerned about the “haves” and “have-nots” in the student body, between which she saw a large divide produced because of inequalities in their access to funding and digital devices. She expressed wanting to “give them a good direction”, “financial help” and a “little bit of guidance whenever they need it”, such that they would be enabled to take “good decisions” and “scholarships… jobs”. She constructed herself as the benevolent representative of the casteless General category, with the power and agency to grant favours to deprived, but deserving, students in the university. Despite this recognition of inequality, in her responses she expressed anxiety about how the politicization of caste on the campus was “becoming too big”, and harming the university by segregating academic citizens into SCs, STs, and OBCs. As a solution, she advocated blinding the assessment by removing “the ‘caste’ column from the application, I think will solve most of the problem” that *the dominant in-group* was experiencing.

The exception to the dominant assertion that quotas should be removed was the criteria for financial need, which was expressed by many. Participants recognized that the economics of competitive access to elite HEIs requires existing financial privilege. A first-generation male academic of the SC category at UI4 (P24) spoke at length about how access to resources was very important for becoming an academic. By way of example, he discussed how postgraduate fellowships offer “proper accommodation, proper food, and also safety and security” along with an “excellent culture of learning”. He emphasized that this was particularly the case for first-generation students from lower caste backgrounds without financial means who would find even the cost of photocopying a book prohibitive.

A very few academics were certain that the reservations policy was of value for social justice. They questioned meritocratic assumptions of fair competition between those positioned unequally, such as in this excerpt from the interview with a male academic from the General category at UI1 (P4).

… when 60% backward class, Dalits, or whoever, or even forward class poor people, if they are not getting the primary quality education, how can you think that they can fight?… They shall remain labourers. Reservation is perhaps the only means to make them empowered.

This person recounted observing how affirmative action policies had led to educated Dalit members enter academia, the civic service, and the police, claiming that when viewed “in totality, it has served the cause of social justice”.

One of the leaders, in a middle management position in quality assurance at UI1 (P10), who held national reservation policies as inviolable, nonetheless extolled that simplistic individualistic measures could address what he constructed as the internalized backwardness of caste. He sketched a deficit portrayal of student self-belief, and stressed that motivation was needed “to harness your potential energy and turn it in the right direction”, contending that the dominant group should act to “counsel such people” “so that they have self-realization… self-retrospection to extract out the latent potential”. Psychological, rather than social, solutions were most prevalently asserted to label those from the reserved categories as “vulnerable” personalities thereby dismissing the structural and cultural discrimination embedded in the fabric of the society. In other instances, educational solutions too seemed unconscious of the socio-cultural. For instance, a female professor within UI1 from the General category (P3) spoke about the importance of institutions offering “bridge courses” for candidates to prove themselves after initial support, rather than taking the approach of “filling the seats [of the reservation quota]… why should we strive for that? Is it a dinner plate?” Clear binary associations linked “weak” and “strong” with those from reserved and unreserved categories, respectively. Any engagement with the privileges of those labeled as strong and legitimate inheritors of social and educational capital, that is, those of the General category, was absent. Deficit constructions of those of the first generation extended across the hierarchy — from before their entrance as students to when they applied as successful post-graduates for academic positions. An associate professor in STEM at UI3 (P14) explained that she was completely against the quota system as it was “hampering the quality because they are not at par with the General category students”. According to her, “the reservation category students” should not be permitted to pursue engineering, implying that doing so posed risk “because engineers are making flyovers, and their skill and intelligence is so much important”. The exclusion of students from reserved category was attributed, by a senior professor at UI3 (P12), to various signs of their mis-fit with the norm, from a “lack of communication skill” to “being non-cosmopolitan”. Some of this had to do with their lack of proficiency in acquiring English language skills which excluded them from gaining access to NMC membership, as discussed earlier in this paper. This further confirmed why many first-generation students were failed from meeting the selection mechanisms that control access to quality education. This academic’s stance on the quota system being a serious hindrance to quality education was underpinned by constructions of deficit capital that reflect the societal bias which should be present in meritocracies.

Participants revealed how casteist attitudes pervaded selection, as shared by a female Humanities professor who recounted an incident which occurred during a doctoral selection committee meeting at UI1 (P7). The first few students were selected based on their results and accepted on the merit list. A number of those from SC groups were placed under the General category in the merit list in accordance with the existing reservation rules where the Court had reiterated that “the unreserved or general category cannot be treated as a de facto quota for upper castes” (Deshpande, 2013, p. 38). An upper caste colleague, ignorant of those rules, exclaimed, “*Ab yeh gobar mitti wale top karne lagenge to hamare bachche kaha jayenge?*” which translates to “if these people who clean dirt become toppers, where would our children go?” This comment reveals casteist discrimination in its associations of Dalits with work related to foraging and cleaning, in addition to characterizing access as a threat to the dominant in-group and the self-interest inherent to the processes of judgment for selection. It is indicative of why meritocracy cannot succeed when hegemonic, exclusionary attitudes towards access persist in those with the powers of selection.

### Intersectional complexities of discourses of inequality

Caste and gender are co-constitutive categories, and as such intersectionality is an important approach to “recover” the marginalized voice of lower caste women (Dhawan, 2017, 2019).^12^ Intersectionality can be used to question the polarization between the sharp binaries of gender (as the upper caste woman) and caste (as the lower caste man) that leaves no space for the lower caste woman. When probed, the narratives of our research participants revealed much entangled interests and assertions in relation to the discourses of inequalities within the university.

A gender person in UI4 (P34) shared how the university campus had “never wanted to look at gender as a unified category but always looked at intersections with gender”. Her experiences as a professor who had initiated a course on gender in her department had reduced her optimism when it came to questions of gender.

… the gender question is never the primary question. So they say, ‘Ok, yeah, but are you gonna complain against a Dalit boy?’ So that becomes more important than whether she’s a Dalit woman who has faced both upper caste patriarchy as well as her own community’s patriarchy.

She believed that female students of lower castes, unlike the conditions created for the new Indian woman and for their male counterparts, suffer greater discrimination that is rendered invisible by the politics of representation. Scholars have referred to the “casteist and exclusionary” nature of the students’ movements on campus which have ignored Dalit women’s interests (see for instance, Committee, 2000). They have asserted that it is imperative for Dalit women to foreground their rights and demand a re-examination of the caste-gender complexities by questioning the largely mutually exclusive groups — upper caste women and Dalit men — to make possible such coalitions (ibid).^13^

A professor from SC category at UI4 (P24), specializing in Dalit Studies, shared how “most of the faculty members, they work with this kind of an elitist mindset” and those from upper-caste background were “reluctant to guide the students from the marginal communities, especially the first-generation students”. In this own academic practice, he tried to do “some kind of justice to the students who come from these kinds of background”. However, he pointed to the continual societal influence which reduced opportunities for access. One was the claim that male children are given preference to be the first of their family to access higher education. This was an explanation provided for the larger participation rate of first-generation male academics, in addition to the continuation of patriarchal cultures within that peer group. He described this as.

a kind of clash of interests… some of the Dalit girls, they told me personally that, ‘Sir, there is a gender discrimination among Dalits… the Dalit boys, they always like the Black-Americans.’ They follow black patriarchy, right? So there is a commonality between the Black patriarchy and Dalit patriarchy.

In another instance, a professor in the Humanities at UI1 (P7), who was very vocal in her protest against injustice in the university, believed “in putting in my effort because somebody who comes after me will have a little courage to go a little ahead of the point that I have reached”. She spoke of an incident that she believed reflected the complex realities of relations of power in universities. When a Dalit male PhD scholar was accused of plagiarism by his upper caste female supervisor, fears of “Dalit atrocities claims” infringed on disciplinary processes leading university authorities to be slow to respond or be seen to choose “sides”. The outcome was changing the supervisors to ones which graduated the student. She felt that “this is one side of the story where the powerful and strong Dalits who have got connections and they have connivance with the upper caste people”. What is also of interest is that an upper caste male colleague laid the blame on the female supervisor for taking on the Dalit student in the first place, using the discriminatory idiom, “*koyla haath me lenge to kala to hoga hi*^14^ and why do you want your palms to be black?”.

Some female participants felt that the approach to the inclusion of those with other markers of difference, such as from caste or religion, had the effect of reducing the emphasis on equality for women. One female academic in STEM at UI3 (P14) adopted the myth of meritocracy uncritically as she ridiculed the students admitted on quota, saying that “they would be looking at teachers as if they are being taught in Urdu or Pharsi (Persian language)! They don’t know anything”. The discourses she used reflected her perceptions that the category of “woman” is homogeneous, and hence was ignorant of the ways in which “caste and gender subjectivities were shaped by intersection of both privilege and oppression, making way for positions of domination and subordination to work in complex ways” (Dhawan, 2019, p. 135). Dalit women’s organizations in post-Mandal India had thrown crucial political questions at feminism for “neglecting their experiences and universalizing the Hindu upper caste, middle class woman as the normative subject of feminism” (ibid). In case of HEIs, the privilege of upper castes, such as the professor referred to above, is that their traditional caste capital entitles them to remain “unmarked” and casteless in the discourse of the new Indian woman. She may then find it strategic to highlight her gender identity for its salience or conceal her caste identity when it leverages privileges, consolidating her as a member of the rising middle class in partnership with counterpart middle class men.

### Epistemological struggles: denial and misrecognition

While the previous section discussed institutional spaces not being friendly to students in reserved categories, also evident in the data was continued denial of discrimination and exclusion, and a lack of reckoning with the misrecognition of social groups with markers of difference.

One professor in Health Sciences from UI3 (P15) shared her concern about academics from reserved categories. As an upper caste academic, who did not support reservation of any kind, she suggested that there may be a developmental culture emerging from, or affinity empathy held by, those of the younger generation, including students, for staff who had been granted access through the quota process.

If a non-quota faculty teaches badly, they [students] revolt. And maybe for quota faculty, they are more sensitive and don’t always revolt. They think it’s better not to complain since the faculty is appointed through quota.

Of interest is that this respondent did not express surprise that students were privy to this knowledge about staff members.

Wide and divisive social and intergenerational gaps between those in academic communities were described as having created an “epistemological struggle in the university space”, an apt insight by a first-generation male professor from the SC category at UI4 (P24). In this section, we discuss the nature of responses to such struggles as they were expressed by the dominant in-group’s denial of systemic and social injustice.

A dominant narrative, most strongly asserted from participants at the institute of technology UI3, was that there was no discrimination, especially on the basis of caste, between academic staff. As such, we offer insights specifically from that HEI. For instance, a woman leader (P23) who was a member of the Board of Governors explained that.

once the students come into the institute, they largely start from the same blank page. Within the institute… I can say about my institute with lot of confidence, that you will not see any kind of discrimination et cetera, in terms of various communities or castes et cetera. it doesn’t figure in the public discourse in any way at all.

It is possible that the overt focus on issues of gender discrimination from those at the institute of technology was possible because the percentages of female students and academics were very low. Not only did they question the associated gender stereotypes, they supported reservation policy of 14% for female students. This commitment to gender parity was not extended to the under-representation in terms of caste. When it came to the quota system for OBC/ST/SC, 10 out of the 13 respondents at that institution did not support it. Common discourses constructed the latter as a system “diluting the quality of education” as expressed by a female academic (P15). A male academic, an assistant professor in the Humanities (P11), described caste reservations as a “big road block… here in India we are just forcing them to go to higher education by giving them this kind of lolly pops” which he saw as against the public good as “big blow for the country”. In his responses, this respondent not only constructed his colleagues from such social groups as undeserving others, but clearly indicated the entitled position he placed himself and the social grouping to which he identified. We found that even those who saw themselves as sympathetic to the groups previously marginalized, indicated the limits of their commitment to social justice. The sentiment of one male leader, a senior professor in STEM who was associated with UI3 for three decades (P22), echoed by many, was that only one generation should benefit from affirmative action, and that “once they are given, no further”. Many did not recognize reservation as a mechanism of addressing societal inequalities and historic injustices, rather constructing it as unfair that groups other than their own had access to financial privilege by virtue of caste-based quota s. An assumption was that a single generation of recipients of the quota system was sufficient opportunity for success of persons from such groups within India’s stratified society.

Most prevalent in their narratives was a denial of any problems of exclusion or discrimination in their midst, which led to their dismissal and misrecognition of such experiences of their marginalized colleagues. What was most evidently lacking was historic awareness of the country’s constitutional commitments and the university’s role in such transformative change. What was more often repeated were a number of urban legends about the supposed abuse of the quota system. One was that of SC/ST/EWS certificates being bought by the wealthy for access to higher education. A professor from STEM (P15) claimed that,

If you check, you will find the people of remote rural areas or poor tribal families, who really need reservation cannot come to study here. Scheduled Castes or Scheduled Tribes students who are entering HEIs through quota have 4 to 5 houses, all in their family are well established and still using quota to get facilities and there is no dearth of money.

A number of such tropes littered the transcript of an interview with an academic from the Social Sciences, an associate professor who included gender and sexuality issues in her teaching (P19). While on the one hand describing facing “a lot of non-cooperation from male faculty members”, on the other, she declared caste an “obsolete concept”.

With such tropes came expressions of moral outrage voiced by many of the participants across the institutions, which often constructed the speaker as benevolent towards the most vulnerable. For instance, one male academic, who was a professor from the Arts and Humanities at UI1 (P1), saw the caste system as an illness of the system. He suggested an evaluation of the efficacy of the quota process for social justice by calling for an “analysis of the system… to see who all gained and who all didn’t gain from the system. And those who benefitted, are they really empowered? And finally, the society which we wanted to change through this system, did it change at all or not?”.

Anxiety about corruption and complaints about misuse and political tensions around reservation policies were expressed by those in leadership and in academic positions alike. While this may seem aimed at those outside of the institution, it was related to a theme of blame shifting that was enacted on those *within* the academic community too, as we discuss in the next section. This can be seen as symptomatic of the denial and misrecognition discussed in this section, which confirms Deshpande’s (2013) analysis that caste has become recognized only as a source of vulnerability and deficit in the rise of the NMC, discussed in the introduction to this chapter. The consequence of this on the cultures of critical consciousness of academic staff, and in turn their sense of responsibility for enacting change, is considered in the next section.

### Blame shifting: cultures of apathy and the externalization of responsibility

The most extreme, fatal consequences of caste discrimination in higher education are publicly known in the stories of suicides of lower caste students such as Rohith Vemula, Chuni Kotal, and Payal Tadvi.^15^ Scholars have shown how these untimely deaths had “renewed [the] fight for visibility for the socially marginalized, for the right to protest and for a capacious notion of social justice” across the country (Arunima, 2017, p. 8, Deshpande, 2016). There were similar reflections around the issue of suicides of university scholars from a majority (11 of 14) of the participants from UI4, which had seen such tragic incidents in the past in their university. A senior male professor from the Social Sciences (P29) who was from the upper castes clearly asserted that “identities like caste are not you know like… the shoes they leave outside the lab when they come in… it’s there on their bodies”. He was especially critical of the Science schools with whom he had tried to initiate conversations on this sensitive issue because “they are sort of impervious to these larger changes in our society”. He shared that “many of the Science faculty… respond with indignation” when it is pointed out that “there is discrimination in the way you deal with Dalit students in the classrooms and in the labs, the way you administer your PhD programme”. Such discriminatory perceptions were expressed by a female academic at UI3, an associate professor in STEM (P14). She attributed such ill-health not to higher education or social injustices, but to the deficit of those groups.

I am dead against it… The SC, ST students are entering IITs and they couldn’t compete, they are committing suicide and the whole thing is breaking down.

Perspectives such as these were shared often without the respondent exhibiting any self-consciousness, of how their constructions of the issues at hand or the students or colleagues they were describing, may be received. This may be because it was an accepted and commonly held discourse within that institution. Associated with this was the recurring theme of shifting of blame to students and academics from marginalized backgrounds, as an externalization of responsibility which was often attributed by participants to cultures of apathy prevalent within the larger Indian higher education system. An example of blame shifting underpinned by misrecognition is this male leader from UI3 (P21) who attributed the mental ill-health endured by those marginalized entrants to government affirmative action policies. He stated, “Instead of equipping them properly, government make their entry through quota. And after they enter, they become mentally unstable, or commit suicide. Even if they continue initially, they finally drop out”.

Similarly, a female academic in STEM at the same institution (P14) expressed that “because the government is compromising, everyone is compromising, and finally when the result comes it is the degradation of the whole system”. When asked during the interview whether she perceived that reserved category students felt humiliated by such associations, she intimated that within the classroom “sometimes they share and say that they feel bad when they are told all that”. Her advice, albeit benevolent, reproduced a culture of performativity to meet the expectations of the gaze of the dominance in-group.

You better show them; you get good grades. Prove to them that you are capable and it is not only because of your reservation you are here. If you you are capable, you crack an exam and show them.

In their bid to mask the structural hierarchies and violence prevalent in higher education institutions, these were attempts of privileged academics to blame, dismiss, repudiate, and marginalize the discriminatory experiences of students from reserved categories.

A first-generation male academic in the Arts and Humanities at UI4 (P24) saw a correlation between the socio- economic background of students and suicide rates in higher educational institutions. He critiqued his colleagues as operating with an “elitist mindset” that sustained an institutional culture of apathy towards such students, where their teachers did not invest in nor sustain students’ hope. He believed the untransformed casteist culture fed into such unwillingness to support SC or ST students.

I noticed in our university, especially in Social Sciences, that most of the faculty from the upper caste background, with a middle-class identity, they were not willing to… were reluctant to guide the students from the marginal communities, especially the first-generation students.

While most of what he described was at the interactional level, he was in favour of focusing on systemic and structural issues which could lead to institutional plurality, instead of targeting individuals for social mobility or concentrating on socio-cultural identities.

Against the norm, a small minority of our respondents saw the experience of belonging of academic citizens as sitting within their sphere of influence. This first-generation male academic (P24) shared,

I feel, at least in my capacity, I try to do some kind of justice to the students who come from these kinds of background… even losing one life is also precious… [the] institution should take the responsibility to sustain them once they enter*.*

He described how the dominant culture made it is easy for colleagues to blame the victim, not only in their thinking but also practically, such as through approaches to closing cases of caste or gender discrimination. Another participant (P29) spoke about how the aftermath of a suicide had been handled. He was concerned that in the face of a “very politically charged” situation where “various entities were trying to take advantage”, there had not been adequate approaches to seek solutions to ensure it does not happen again.

nobody has talked about any systemic issues that have led to that death. Was there any systemic issue, were there any structural issues, which need to be addressed? Gap which needs to be plugged…to prevent something like that from happening again.

He also spoke about a lack of accountability about issues where exclusionary practices of bullying may have played a part (“individuals have not gone away. They have survived all the attacks”), and that the status quo of the conditions which lead to such mental ill-health “remains the same. So you have not… actually done any service to any possible institutional changes”. Similarly, another assistant professor (P31) in Health and Life Sciences at the same institution (UI4) discussed the difficulties such students face in finding willing supervisors because staff “have an attitude that I will not take any students who is coming under the reserved category”.

A “gender person” who was an associate professor (P33) noted that the effects of the exclusionary conditions were pathologized as atomized at the individual level, rather than being informed by the collective resources of those within the university who could contribute to more social models of understanding transformation systemically.

[on] this campus suicide is a contested fraught concept and even for people who’ve been involved and been thinking about it, [it’s] difficult for us to articulate that question. But they would have… a medicalised question.

## Conclusion

Within this paper, we have located our argument in the discourse of the rise of the New Middle Class (NMC) in India from the 1990s (Fernandes & Heller, 2006; Sheth, 1999). This echoes production patterns of social stratification in contexts of high participation in higher education (Marginson, 2016) through to developing contexts as diverse as China (Ding et al., 2021; Tsang, 2013), Chile (Chiappa & Perez Mejias, 2019), and Russia (Konstantinovskiy, 2017). What we found was that rather than practicing social justice, the enactment of policies of affirmative action have altered public universities into producers of the NMC even while they claim to be expansive and inclusive. While the entry of subalterns is framed as a brand of the NMC modernity, mobility, and development, any assertion or retention of their gendered, caste, or minority identities are simultaneously repudiated and misrecognized. It is the “unmarked”, “universal” and dominant fraction of the NMC who desire control, not always with success, to define the limits of legitimate participation of the subalterns in the university space, and in turn the world beyond.

While the constitutional obligation of social justice has spurred the rise of NMC in public universities, it has simultaneously revealed a shamelessly pretentious commitment to social justice (Visvanathan, 2000). In a desperate bid to preserve their hegemonic positions in the university and the anxiety to hold over Indian knowledge formation and social formation, the privileged upper castes (including those within Women’s Studies) have failed the institution’s social contract of pursuing the public good. Undoubtedly, the subject of caste was considerably the most difficult and awkward for participants from the dominant in-group to discuss in this study. The neoliberal discursive jargon of “quality” and merit gelled well with the exclusionary upper caste discourse of the General category, especially for those who seemed in denial of the systemic issues of caste inequality and strove to render it invisible within the institution of higher education. They adopted the myth of meritocracy uncritically, and related deficit discourses of marginalized groups to both the abuse of constitutional policies and to cases of mental ill-health and suicide. Some upper caste participants did speak self-critically about their positionality, expressing “anguish”, “guilt” and the vulnerabilities of negotiating their own privileges. However, the translation of such individualized responses to structural change was flimsy. In addition, the nature of participants’ orientation to social justice varied — from those that realized equity was important, to those who positioned education as an unproblematic benevolent force for the enlightenment of those from supposedly deficit communities, to those who stratified applicants uncritically into the un/deserving using various arguments to substantiate their flawed interests.

Of all to emerge, it is perhaps the figure of the subaltern woman (Bhatt et al., 2010) in the NMC university space that is the most significant and progressive rupture from the higher educational past, defined through oppressive intersections of gender and caste. While her arrival is claimed as inclusive, apolitical and expansive on the part of the NMC, the aggressive, successful, and confident figure of the subaltern woman produces anxieties and struggles for “hegemony between the middle class and subaltern classes” (ibid, p. 148). What our analysis confirms is that the figure of the subaltern woman can be seen as useful for the dominant in-group within higher education, however only as long as that figure continues to define a particular form of justice and progress, and does not assert subaltern identities. She is thus “both incorporated into NMC hegemonies as well as excluded from them”, in patterns recognized previously by Bhatt et al. (2010, p. 147). Upper caste women from the “general” category have the privilege in the NMC of claiming themselves as casteless academics in universities, and make strategic alliance with upper caste male academic staff to affirm delusions of a post- caste culture. However, for subaltern academic women, despite having acquired their jobs and positions in universities through their hard work, determination, and against the odds, this does not necessarily translate into empowerment. We are thus reminded of our beginning discussion of the reception of Dr. Murmu’s position and how when she occupied her assigned authority as an academic, it produced unease generally, including among upper caste female colleagues. In such ways, the limits of the NMC inclusivity are marked within the academy — a major problematic for plurality and democracy in this powerful institution.

## Footnotes

1 Dr. Dhawan and Dr. Belluigi convened the colloquium ‘TRANSFORMATION FOR SUSTAINABILITY? Gender and its intersections within participation in higher education’ on 10–11 of February 2020 at the School of Women’s Studies, Jadavpur University, Kolkata, India. The concept note and book of abstracts are available at https://pure.qub.ac.uk/files/201453945/Colloquium_Booklet.pdf.

2 Elaborating the caste-based critiques of gender, along with Ambedkar, feminists have also rediscovered the forgotten legacies of anti-caste leaders from colonial India such as Jyotiba Phule and Periyar (Chakravarti 2002; Rege 1998, 2006).

3 The 1980 report of the Mandal Commission recommended that, in addition to the 23 percent of various positions reserved for the Scheduled Castes and Scheduled Tribes, a further 27 percent be reserved for the Other Backward Classes. The reservations of 15% for SCs and 7.5% for STs were implemented in 1950 when the Indian Constitution reserved seats in public sector employment, educational institutions, and government representative bodies (Galanter 1989). The reservation of 27% for OBCs was implemented in 2006 (John 2012).

4 We recognize the problematic terminology inherent to these categories. However, to respond appropriately to the issue, we utilize the state-mandated terminology.

5 This data was obtained through multiple Right to Information (RTI) applications filed with individual institutes and the Ministry of Human Resources Development, Government of India from 2016 to 2018. Also see Joshi and Malghan (2017). The Right to Information (RTI) Act of 2005 is an act of the Parliament of India which sets out the rules and procedures regarding citizens’ right to information mandating timely response to their requests.

6 The term ‘mandalization’ is from the Mandal Commission, named after its President, B. P. Mandal. The Commission was appointed in 1978 to determine the criteria for defining which groups were socially and educationally marginalized, and to recommend steps to be taken for their advancement. The 1980 report of the Mandal Commission made important recommendations (refer footnote 3) which were shelved by successive governments probably in order to avoid arousing inter-caste tensions. It was finally on 7 August 1990 that V. P. Singh, the then Prime Minister of India, announced that the recommendations would be implemented.

7 Endogamy is the crucial tool for the manifestation and perpetuation of caste and gender subordination. It is a rule enjoining marriage within a specified caste-based group, with the marriage then termed endogamous marriage. Refer to Chakravarti (2002). However, women within such dynamics should not be constructed as passive followers of norms. They have continuously contested, rejected, and redefined the ‘legitimate’ in marriage (for instance, see Sen et al., 2011 for more).

8 Belluigi (2020, n.p.) refers to social justice in higher education operating as an empty signifier, with its usable definition being “to remedy the injustices of society through higher education”.

9 This study was part of the project titled ‘Transformation towards Sustainability in Higher Education: Interactional Dynamics in Gender and Intersectionality,’ funded through RUSA 2.0 (Rashtriya Uchchatar Shiksha Abhiyan) of the Ministry of Human Resource Development, Government of India. Ethical approval was awarded by the Research Ethics Committee of Queen’s University Belfast.

10 We use Henderson’s (2019) term where she uses the colloquial expression ‘gender person’ to refer to those academics who research and/or teach gender but whose primary affiliation is not women’s/gender studies. In India, because of the lack of academics tenured in women’s/gender studies it is quite usual for gender persons to give voluntary services to women’s studies and they also act as research collaborators with academics in centres or departments of women’s/gender studies.

11 We have been careful to indicate our awareness of the limitations of this in this papers’ analysis; in that, it gives a stronger dominance to that in-group. Of interest is that there was more diversity to the questionnaire responses in the first phase of our study, which have informed other papers.

12 There have been varied debates in Indian feminist scholarship on the importance and applicability of intersectionality in the Indian context. Refer to Menon (2015) and John (2015).

13 The ‘Dalit’ caste is equivalent to the constitutional category of Scheduled Caste (SC).

14 This translated to ‘if you take coal in your hands, your hands will surely get dirty,’ a casteist comment using metaphors of coal and dirt for Dalits.

15 Rohith Vemula was a PhD scholar at the Central University of Hyderabad who ended his life on 17 January 2016 owing to a sense of deep isolation which resulted in a strong students’ movement across colleges and universities in India. Chuni Kotal was the first woman graduate from ST community to commit suicide in 1992 at the age of 27, from what many believe were her experience of years of casteist harassment in a university in West Bengal. Payal Tadvi, another ST woman, committed suicide in 2019 from enduring casteist harassment from her seniors at a National Medical College in Maharashtra. There have been strong student movements in different parts of the country on such issues. For more details, see Arunima (2017), Chaudhuri (2019), and Deshpande (2016).

## References

[CR1] AISHE. (2020). *All India Survey on Higher Education 2019–20*. Ministry of Human Resource Development, Department of Higher Education, Government of India, New Delhi. Retrieved May 13, 2021 from http://aishe.nic.in/aishe/reports

[CR2] Alam, J. (1999). Is caste appeal casteism? Oppressed castes in politics. *Economic and Political Weekly*, *34*(13), 757–761. https://www.jstor.org/stable/4407794

[CR3] Archer L (2007). Diversity, equality and higher education: A critical reflection on the ab/uses of equity discourse within widening participation. Teaching in Higher Education.

[CR4] Arunima, G. (2017). Thought, policies and politics: How may we imagine the public university in India? *Kronos, 43*(1), 165–184. 10.17159/2309-9585/2017/v43a11

[CR5] Belluigi, D. Z. (2020). *The challenges of social justice in and of higher education*. Public talk at Jadavpur University, India. January 2020. Audio retrieved June 20, 2021, from https://pure.qub.ac.uk/en/activities/the-challenges-of-social-justice-in-and-of-higher-education

[CR6] Belluigi, D. Z., Dhawan, N. B., & Idahosa, G. E. (2020). *Sustainability is based on the faith we have towards the work that we are doing": The conditions of academic citizenry in South Africa and India*, paper presented at Africa Knows! It is time to decolonise minds, 2–4 December, Conference of the Leiden African Studies Association. Retrieved January 23, 2021, from https://nomadit.co.uk/conference/africaknows/paper/57778

[CR7] Belluigi, D. Z. & Meistre, B. A. (2021). Authoring author-ity in transition? The ‘Counter // Narratives of Higher Education’ Project. Video of paper for the conference *Visualising Social Changes: Seen and Unseen* of the International Visual Sociology Association Annual Conference. Retrieved March 13, 2021, from https://youtu.be/mnQIBiGM9Uc

[CR8] Bengtsen S, Barnett R (2016). Confronting the dark side of higher education. Journal of Philosophy of Education.

[CR9] Bhatt A, Murty M, Ramamurthy P (2010). Hegemonic developments: The new Indian middle class, gendered subalterns, and diasporic returnees in the event of neoliberalism. Signs.

[CR10] Bhattacharyya, I. (2020). Collective caste hatred stuns Bengal academia, support pours in for JU Prof Maroona Murmu, 8 September. Retrieved March 15, 2021 from https://thewire.in/rights/maroona-murmu-jadavpur-university-collective-caste-hatred-bengal-academia

[CR11] Bhushan, S. (2019). A dithering higher education policy. *Economic and Political Weekly, 54*(24), 12–14. Retrieved July 23, 2020, from https://www.epw.in/journal/2019/24/commentary/dithering-higher-education-policy.html

[CR12] Brennan J, Naidoo R (2008). Higher education and the achievement (and/or prevention) of equity and social justice. Higher Education.

[CR13] Brim, M. (2020). *Poor queer studies: Confronting elitism in the university*. Duke University Press.

[CR14] Carnoy M, Dossani R (2013). Goals and governance of higher education in India. Higher Education.

[CR15] Chadha, G & Achuthan, A. (2017). Feminist science studies: Intersectional narratives of persons in gender-marginal locations in science. *Economic and Political Weekly, LII*(17), April 29. Retrieved June 25, 2019, from https://www.epw.in/journal/2017/17/review-womens-studies/feminist-science-studies.html

[CR16] Chakravarti U (2002). Gendering caste through a feminist lens.

[CR18] Chaudhuri S (2019). On making noise: Hokkolorob and its place in Indian student movements. Postcolonial Studies.

[CR19] Chiappa R, Perez Mejias P (2019). Unfolding the direct and indirect effects of social class of origin on faculty income. Higher Education.

[CR20] Chowdhury, S. (2020). Jadavpur University teacher files police complaint, 9 September. Retrieved March 16, 2021, from https://www.telegraphindia.com/west-bengal/calcutta/jadavpur-university-teacher-files-police-complaint/cid/1791582

[CR21] Committee, J. A. (2000). Negotiating gender and caste: A struggle in Hyderabad Central University. *Economic and Political Weekly,**35*(43/44), 3845–3848. https://www.jstor.org/stable/4409892

[CR22] Corbridge S, Harriss J (2000). Reinventing India: Liberalization, Hindu nationalism and popular democracy.

[CR24] Datla K (2013). The language of secular Islam: Urdu nationalism and colonial India.

[CR25] Deshpande, S. (2013). Caste and castelessness: Towards a biography of the ‘General category’. *Economic and Political Weekly, 48*(15), 32–39. https://www.jstor.org/stable/23527121

[CR26] Deshpande, S. (2016). The public university after Rohith-Kanhaiya. *Economic and Political Weekly, 51*(11), 32–34. Retrieved May 21, 2021, from https://www.epw.in/journal/2016/11/university-under-siege/public-university-after-rohith-kanhaiya.html

[CR27] Dhareshwar, V. (1993). Caste and the secular self. *Journal of Arts and Ideas, 25–26*, 115–126. Retrieved November 12, 2020, from http://cscs.res.in/dataarchive/textfiles/textfile.2007-08-16.9861664259/file

[CR28] Dhawan NB (2010). The married ‘new Indian woman’: Hegemonic aspirations in new middle class politics?. South African Review of Sociology.

[CR29] Dhawan NB (2017). From ‘fire-brand’ to ‘water-brand’: The caste politics of Uma Bharati. Asian Journal of Women’s Studies.

[CR30] Dhawan, N. B. (2019). The Hindutva politics of Uma Bharati: Challenges to women’s movements, in Banerjee, S and Ghosh, N. eds, *Caste and Gender in Contemporary India: Power, Privilege and Politics*. Routledge.

[CR31] Dhawan, N. B. (2011). The ‘legitimate’ in marriage: Legal regulations and social norms, In Sen, S. et. al. (Eds.), *Intimate Others: Marriage and Sexualities in India*. Stree.

[CR32] Dhawan, N. B., Belluigi, D. Z., & Idahosa, G. E. (2020). Gender equity in Indian higher education: Vision of institutional leadership, paper presented at the International Conference on Gender, Language and Education: *Equality and Diversity Issues in Asia and Beyond,* hosted by The Education University of Hong Kong, 2–4 Dec.

[CR33] Dhawan, N., Belluigi, D. Z., & Idahosa, G. E. (2021). Disrupting the ‘new middle class’ space of Indian public universities. Paper presented at the *International Conference on Globalisation of Professional Legal Education: Constitutional Conspectus*, 3 April 2021, organized by the School of Law, Bennett University, Greater Noida, India.

[CR35] Ding, Y., Wu, Y., Yang, J., & Ye, X. (2021). The elite exclusion: Stratified access and production during the Chinese higher education expansion. *Higher Education, 82, 323-347. *10.1007/s10734-021-00682-y 10.1007/s10734-021-00682-y PMC8023350 33840818

[CR36] Dirks N (2001). Castes of mind: Colonialism and the making of modern India.

[CR37] Fernandes, L. (2004). The politics of forgetting: Class politics, state power and the restructuring of urban space in India. *Urban Studies, 41*(12), 2415–2430. Retrieved 15 November 2021, from https://www.jstor.org/stable/43197064

[CR38] Fernandes L, Heller P (2006). Hegemonic aspirations: New middle class politics and India’s democracy in comparative perspective. Critical Asian Studies.

[CR39] Galanter M (1989). Law and society in modern India.

[CR40] Ghosh, H. (2020). *Jadavpur University professor faces casteist abuse for commenting on exams during COVID*, 5 September. Retrieved March 16, 2021, from https://thewire.in/rights/jadavpur-university-professor-faces-casteist-abuse-for-commenting-on-exams-during-covid

[CR41] Henderson E (2019). On being the ‘gender person’ in an academic department: Constructions, configurations and implications. Journal of Gender Studies.

[CR42] John, M.E. (2015). Intersectionality: Rejection or critical dialogue. *Economic and Political Weekly,* 50 (33), 15 August. https://www.epw.in/journal/2015/33/discussion/intersectionality.html

[CR43] John ME (2012). Gender and higher education in the time of reforms. Contemporary Education Dialogue.

[CR44] Joshi, S and Malghan, D. (2018). Why are there still such few SCs, STs and OBCs at IIMs?, 18 January. Retrieved March 31, 2021, from https://thewire.in/caste/iim-sc-st-obc-diversity

[CR45] Joshi, S and Malghan, D. (2017). IIMs have a dismal diversity record — and the new bill is not going to fix that, 16 March. Retrieved March 31, 2021, from https://thewire.in/rights/social-diversity-deficit-iim

[CR46] Kar, A. (2020). *They reduce me to a “meritless” Adivasi: Maroona Murmu Jadavpur Associate Professor*, 10 September. Retrieved March 15 2021, from https://caravanmagazine.in/interview/maroona-murmu-adivasi-associate-professor-on-facing-discrimination

[CR47] Konstantinovskiy DL (2017). Expansion of higher education and consequences for social inequality (the case of Russia). Higher Education.

[CR48] Krishnan S (2017). The engineering of India’s middle-class politics. Contemporary South Asia.

[CR49] Krishnan, S. (2014). The political economy of India’s tertiary education: Persistence and change. *Economic and Political Weekly* XLIX 15, 63–70. Retrieved March 12, 2020, from https://www.epw.in/journal/2014/11/special-articles/political-economy-indias-tertiary-education.html

[CR50] Kumar, U. (2016). The university and its outside. *Economic and Political Weekly*, 51(11): 29–31. Retrieved March 16, 2020, from https://www.epw.in/journal/2016/11/university-under-siege/university-and-its-outside.html

[CR51] Louis, P. (2005). Historicity of Dalit connection. In Teltumbde, A. (ed), *Hindutva and Dalits: Perspectives for Understanding Communal Praxis*. Samya.

[CR52] Marginson S (2016). The worldwide trend to high participation higher education: Dynamics of social stratification in inclusive systems. Higher Education.

[CR54] Menon, N. (2015). Is feminism about “women”? A critical view on intersectionality from India. *Economic and Political Weekly,* 50 (17), 25 April. https://www.epw.in/journal/2015/17/perspectives/feminism-about-women.html

[CR55] Mohanty CT (2003). “Under Western eyes” revisited: Feminist solidarity through anticapitalist struggles. Signs.

[CR56] Murmu, M. (2020). Inefficacious affirmative action and social exclusions in higher education. Paper presentation at the colloquium ‘TRANSFORMATION FOR SUSTAINABILITY? Gender and its intersections within participation in higher education’, Jadavpur University, 10–11 February. Retrieved November 12, 2020, from https://pure.qub.ac.uk/files/201453945/Colloquium_Booklet.pdf

[CR57] Nair, J. (2017). The provocations of the public university. *Economic and Political Weekly, 52*(37), 34–41. Retrieved May 12, 2020, from https://www.epw.in/journal/2017/37/perspectives/provocations-public-university.html

[CR58] NEP (2020). *National Education Policy*, Ministry of Human Resource Development, Government of India. Retrieved December 15, 2020, from https://www.education.gov.in/sites/upload_files/mhrd/files/NEP_Final_English.pdf

[CR59] Nisbett N, Gooptu N (2013). Youth and the practice of IT enterprise: Narratives of the knowledge society and the creation of new subjectivities amongst Bangalore’s IT aspirants. Enterprise Culture in Neoliberal India: Studies in Youth, Class, Work and Media.

[CR60] Rao, A. (2012). Caste and gender in Sen. S. et. al, eds. *Mapping the Field: Gender Relations in Contemporary India*. Stree.

[CR61] Reay D, Arday J, Mirza HS (2018). Race and elite universities in the UK. Dismantling race in higher education.

[CR62] Rege, S. (2006). *Writing caste, writing gender: Dalit women’s testimonies*. Zubaan.

[CR63] Rege, S. (1998). Dalit women talk differently: A critique of ‘difference’ and towards a Dalit feminist standpoint position. *Economic and Political Weekly, 33(*44), WS39-WS46. https://www.epw.in/journal/1998/44/review-womens-studies-review-issues-specials/dalit-women-talk-differently-critique

[CR64] Roundtable India (2020). *Statement of solidarity for Dr. Maroona Murmu from the Faculty of Presidency University*, 8 September. Retrieved February 15, 2021, from https://roundtableindia.co.in/index.php?option=com_content&view=article&id=10000:statement-of-solidarity-for-dr-maroona-murmu-from-the-faculty-of-presidency-university&catid=129&Itemid=195

[CR66] Sancho, D. (2013) Aspirational regimes: Parental educational practice and the new Indian youth discourse. In Gooptu N (ed.) *Enterprise Culture in Neoliberal India: Studies in Youth, Class, Work and Media*. Routledge.

[CR67] Sancho D (2015). Youth, class and education in urban India: The year that can break or make you.

[CR69] Sen, S, Biswas, R., & Dhawan, N. (2011). *Intimate others: Marriage and sexualities in India*. Stree.

[CR70] Seth, S. (2015). Higher education and the Indian social imaginary. *American Historical Review*, *120*(40), 1354–1367. 10.1093/ahr/120.4.1354

[CR71] Shaguri, O. R. (2013). *Higher education in India access, equity, quality.* EAN World Congress Scholar. Global Access to Postsecondary Education. Retrieved November 14. 2020, from https://docplayer.net/6311022-Higher-education-in-india-access-equity-quality-obadya-ray-shaguri.html

[CR73] Sheth, D. L. (1999). Secularisation of caste and making of new middle class. *Economic and Political Weekly* 34, 34/35: 2502–10. Retrieved June 12, 2019, from https://jan.ucc.nau.edu/~sj6/epwshethmclass1.htm

[CR74] Singh, D. (1991). Protest movements in India. *The Indian Journal of Political Science*, *52*(4), 448–57. https://www.jstor.org/stable/41855582

[CR75] Subramanian A (2019). The caste of merit: Engineering education in India.

[CR76] Thomas, R. (2020). Brahmins as scientists and science as Brahmins’ calling: Caste in an Indian scientific research institute. *Public Understanding of Science*: 1–13. 10.1177/0963662520903690 10.1177/0963662520903690 32036740

[CR78] Tsang EYh (2013). The quest for higher education by the Chinese middle class: Retrenching social mobility?. Higher Education.

[CR79] Upadhya C (2016). Engineering equality? Education and im/mobility in coastal Andhra Pradesh, India. Contemporary South Asia.

[CR80] Visvanathan, S. (2000). Democracy, plurality and Indian university. *Economic and Political Weekly,* 35(40): 3597–3606. https://www.epw.in/journal/2000/40/special-articles/democracy-plurality-and-indian-university.html

[CR81] Wilby, M. (2020). An irreversible other. Video artwork 6 min 22 seconds. Retrieved January 30, 2020, from https://counternarrativefilm.wixsite.com/counter/an-irreversible-other

[CR82] Wire (2019). *Less than 3% of all faculty members at IITs are SC/ST*, 2 January. Retrieved February 15, 2021, from https://thewire.in/education/less-than-3-of-all-faculty-members-at-iits-are-sc-st

[CR83] Wire (2017). *Scholars urge IIMs to address their serious diversity problem*, 25 August. Retrieved February 15, 2021, from https://thewire.in/education/iim-directors-diversity-problem

[CR84] Xu, C. L. (2020). Tackling rural-urban inequalities through educational mobilities: Rural-origin Chinese academics from impoverished backgrounds navigating higher education. *Policy Reviews in Higher Education,* 1–24. 10.1080/23322969.2020.1783697

